# Uptrend in global managed honey bee colonies and production based on a six-decade viewpoint, 1961–2017

**DOI:** 10.1038/s41598-022-25290-3

**Published:** 2022-12-09

**Authors:** Bernard J. Phiri, Damien Fèvre, Arata Hidano

**Affiliations:** 1 Ministry for Primary Industries, Wellington, New Zealand; 2 Department of Biochemistry, University of Otago, Dunedin, 9054 New Zealand; 3 AbacusBio Ltd, Dunedin, 9016 New Zealand; 4 London School of Hygiene and Tropical Medicine, London, UK

**Keywords:** Agroecology, Ecological epidemiology, Population dynamics

## Abstract

We conducted a retrospective study to examine the long-term trends for the global honey bee population and its two main products: honey and beeswax. Our analysis was based on the data collected by the Food and Agriculture Organization of the United Nations from 1961 to 2017. During this period, there were increases in the number of managed honey bee colonies (85.0%), honey production (181.0%) and beeswax production (116.0%). The amount of honey produced per colony increased by 45.0%, signifying improvements in the efficiency for producing honey. Concurrently, the human population grew by 144.0%. Whilst the absolute number of managed colonies increased globally, the number per capita declined by 19.9% from 13.6 colonies per 1000 population in 1961 to 10.9 colonies per 1000 population in 2017. Beeswax had a similar trend as the global production per capita reduced by 8.5% from 8.2 to 7.5 kg per 1000 population. In contrast, the global honey production per capita increased by 42.9% at the global level. The global human population growth outpaced that of managed honey bee colonies. Continuation of this trend raises the possibility of having a shortfall of pollinators to meet the increasing consumer demand for pollinated crops. To mitigate these challenges locally driven solutions will be key as influencing factors differed geographically.

## Introduction

Headlines of honey bee colony losses have given an impression of large-scale global decline of the bee population that endangers beekeeping^1,2^ and that the world is on the verge of mass starvation. However, the stories are usually based on research reports limited to one or few countries with observations over a relatively short period of time^3^. A large proportion of cited scientific literature on honey bee mortality originates from Europe and North America^1,4^, creating some sort of publication bias. Further, the research reports are focused on managed honey bees, *Apis mellifera* in particular, with little or no information on non-managed bees^5^. Hence, extrapolation of findings from these reports to the global bee population is somewhat inaccurate. Nevertheless, colony losses have been severe during winter in parts of Europe and North America^6,7^.

Honey bees are socioeconomically important because they play a critical role in crop pollination and produce a variety of products which are vital to many communities and industries^8^. Their importance and contribution to sustainable development has been described by Patel et al.^9^. Bee products that have socioeconomic value include honey, beeswax, propolis, pollen, royal jelly and bee venom^10,11^. Live bees are also bought and sold in form of queens or bulk packaged bees for breeding^12^. In some cases, bees are used as protein sources in human and animal diets^13,14^. Honey is used as medicine, cash crop and critical ingredient in some cultural traditions. Beeswax is not only important in beekeeping for making foundation sheets but also used in more than 300 other industrial processes. These include the manufacture of cosmetics, pharmaceuticals, candles, electronic components, polishes and specialized industrial lubricants^15,16^. Hence, the prospect of an impending or ongoing global decline in the honey bee population implies that there could be wide-ranging socioeconomic impacts.

Therefore, the objective of the current study was to provide an unbiased and quantitative assessment of the beekeeping industry based on long-term temporal and geographical trends globally. To achieve this, we examined changes in the number of honey bee colonies and their two main products: honey and beeswax. Our analysis was based on the data collected by the Food and Agriculture Organization of the United Nations (FAO) over a period of six decades. The dataset does not specify species of the bees, hence, “honey bee” in the current article refers to any bee species but likely to be predominantly *Apis mellifera*.

## Results

### Overview statistics

**Figure 1:**
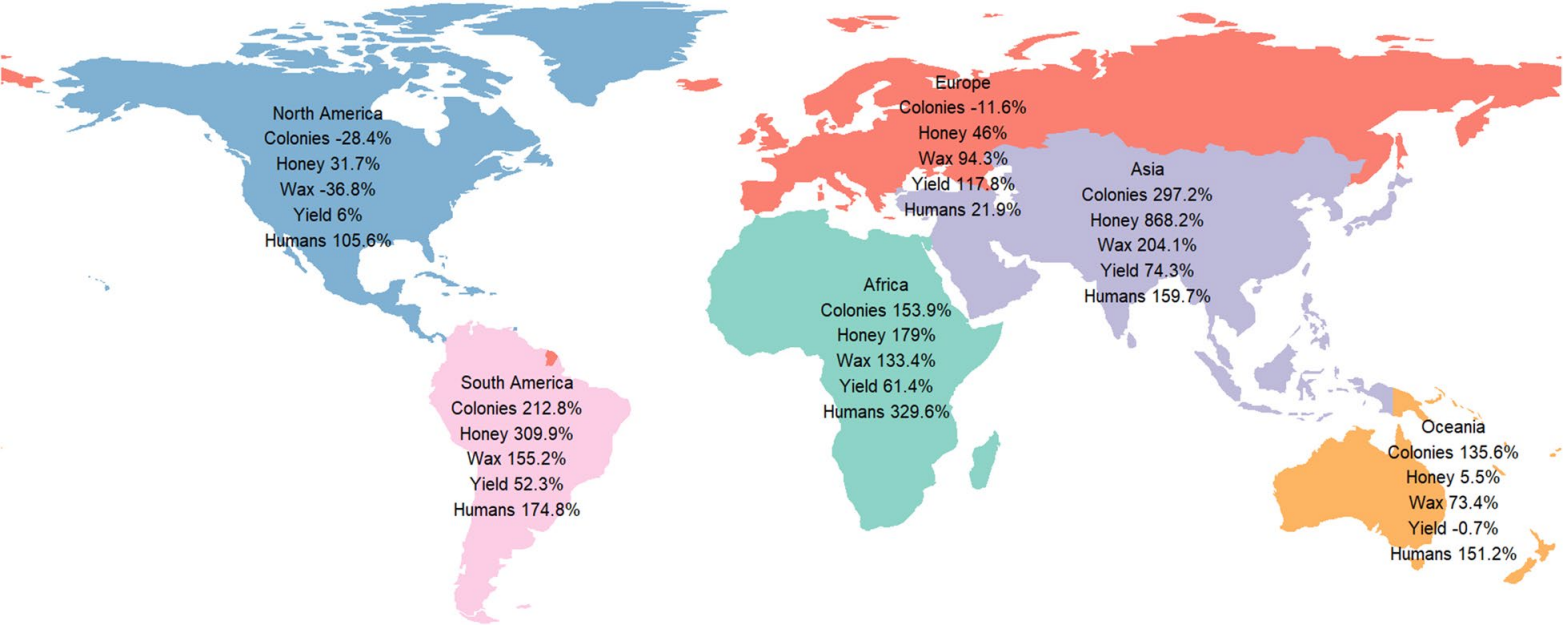
Regional changes, in percentage, of beekeeping variables and human population between 1961 and 2017.

At the global level, all the study variables increased between 1961 and 2017 (Table 1). The number of managed honey bee colonies nearly doubled, honey production almost tripled and beeswax production more than doubled. Concurrently, the 2017 human population was more than twice what it was in 1961. The yield of honey per colony increased by about half over the same period. At the regional level, changes in the study variables ranged from declining to increasing by almost eight-fold (Fig. 1). The number of colonies increased in Asia, South America, Africa and Oceania but declined in North America and Europe. Honey production increased in all regions, remarkably so in Asia and South America. Beeswax production increased in all regions except North America where it declined. The highest increase was in Asia followed by South America and Africa. Europe had the highest improvements in yields of honey per colony followed by Asia and Africa. In contrast, the yields declined marginally in Oceania.

**Table 1:** Overall changes in study variables at the global level between 1961 and 2017.

Variable	1961	2017	Increase (%)
Colonies (000,000)	49.2	91.0	85.0
Honey production (0,000 tonnes)	67.5	189.9	181.0
Beeswax production (000 tonnes)	31.8	68.6	116.0
Honey yield (kg/colony)	11.1	16.1	45.0
Human population (billion)	3.1	7.5	144.0

The number of colonies and volumes of honey and beeswax produced at the regional level are presented as five-year averages both at the start and end of our study period (Table 2). These quantities are also expressed as percentages of the global total. In the early 1960s Europe had the highest number of managed colonies followed by Asia and North America. By the mid-2010s Asia had the highest number of managed colonies followed by Europe and Africa. The ten countries with the highest number of managed colonies during the 1961–1965 and 2013–2017 five-year periods are shown in supplementary Table S1. Almost a third of the global managed colonies were in the Soviet Union and United States in early 1960s. A similar proportion was in India, China and Türkiye by the mid-2010s.

**Table 2:** Global and regional average numbers of colonies and volumes of honey and beeswax produced per annum over the five-year 2013–2017, arranged in descending order of the estimated quantity.

1961–1965			2013–2017		
Region	Quantity (95% CI)	%	Region	Quantity (95% CI)	%
**Colonies (000,000)**					
Global	49.8 (94.5; 101.0)	100.0	Global	88.5 (85.5; 91.5)	100.0
Europe	21.2 (20.9; 21.6)	42.7	Asia	41.2 (39.5; 42.8)	46.5
Asia	11.4 (10.3; 12.4)	22.8	Europe	18.1 (17.3; 18.9)	20.4
North America	7.7 (6.8; 8.6)	15.5	Africa	17 (16.5; 17.4)	19.2
Africa	7.3 (6.8; 7.7)	14.6	North America	6.1 (6.0; 6.2)	6.8
South America	1.7 (1.7; 1.7)	3.4	South America	5.2 (5.1; 5.3)	5.9
Oceania	0.5 (0.5; 0.5)	1.0	Oceania	1.1 (1; 1.2)	1.2
**Honey production (0,000 tonnes)**					
Global	71.0 (66.9; 75.2)	100.0	Global	183.0 (173.0; 193.0)	100.0
Europe	29.5 (26.9; 32.1)	41.6	Asia	83.7 (76.6; 90.7)	45.6
North America	18.1 (17.2; 19.0)	25.5	Europe	39.2 (36.7; 41.8)	21.4
Asia	10.1 (9.4; 10.9)	14.2	North America	23.7 (22.4; 24.9)	12.9
Africa	6.8 (6.6; 7.0)	9.6	Africa	19.1 (17.0; 21.2)	10.4
South America	4.0 (3.6; 4.5)	5.7	South America	14.5 (13.0; 15.9)	7.9
Oceania	2.4 (2.1; 2.7)	3.4	Oceania	3.1 (2.8; 3.4)	1.7
**Beeswax (000 tonnes)**					
Global	31.6 (30.2; 32.9)	100.0	Global	67.2 (65.0; 69.4)	100.0
Asia	11.9 (10.3; 13.5)	37.7	Asia	33.2 (31.3; 35.0)	49.4
Africa	7.4 (7.0; 7.7)	23.3	Africa	16.0 (15.6; 16.4)	23.8
North America	6.6 (5; 8.2)	21.0	South America	8.6 (8.5; 8.7)	12.8
South America	3.5 (3.4; 3.7)	11.2	North America	5.1 (4.8; 5.4)	7.6
Europe	1.8 (1.8; 1.9)	5.8	Europe	3.7 (3.6; 3.8)	5.5
Oceania	0.3 (0.3; 0.4)	1.0	Oceania	0.6 (0.6; 0.6)	0.9

Estimates are accompanied with the 95% confidence interval (CI) and expressed as percentage of the global total.

Europe and North America produced about two-thirds of the global honey in the early 1960s while in the 2010s a third of the honey was produced by China and Türkiye (Table 2). At the country-level, the Soviet Union and United States produced nearly half of the global honey in the early 1960s, in contrast, China and Türkiye produced a third of the honey in the mid-2010s (Table S1). Asia and Africa continued being the major producers of beeswax from the start to the end of the study period. Their combined production increased from about two-thirds in early 1960s to approximately three-quarters of the global total in the mid-2010s (Table 2). India maintained its position as the number one beeswax producing country of the world throughout the study period. It produced a third of the world bees wax in the early 1960s and over three-quarters of it in the mid-2010s (Table S1).

### Honey bee colonies

**Figure 2:**
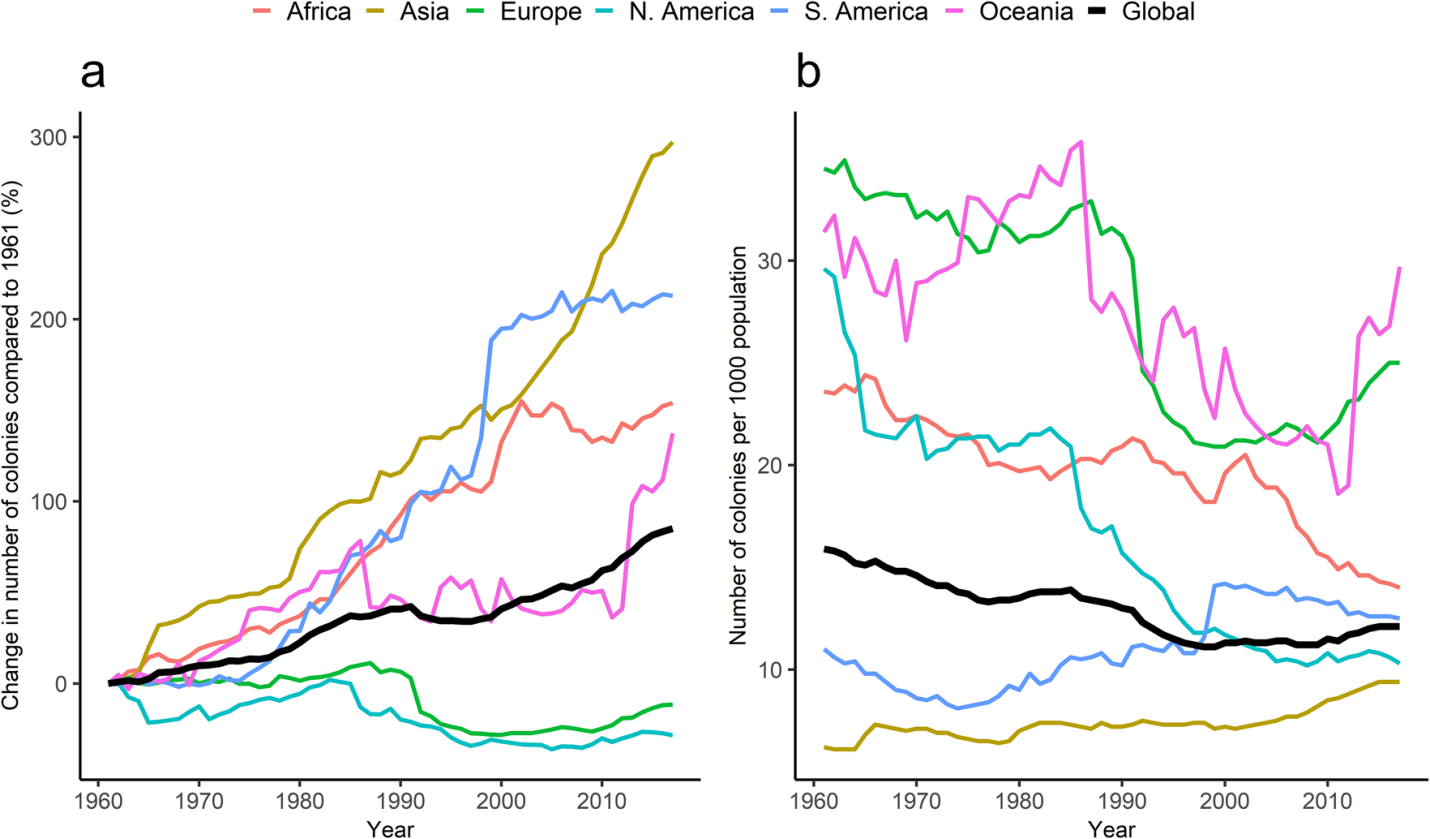
Regional changes presented as percentages of the annual number of managed honey bee colonies compared to 1961 (**a**) and number of colonies per capita (**b**), 1961–2017.

Globally, there was a steady increase in the number of managed honey bee colonies relative to 1961 (Fig. 2a). The upward trends were more consistent in Africa, Asia and South America. Oceania experienced growth from 1961 until the mid-1980s when there was a decline which plateaued until the early 2010s. Thereafter, the number of colonies increased sharply. In contrast, the number of colonies in North America and Europe were persistently fewer than there were in 1961 throughout the study period. However, there were modest increases between 2000 and 2017 of 5.0% (North America) and 23.2% (Europe).

Although the number of colonies increased globally during the period under review, a given number of colonies served a larger human population with time, as indicated by the slope of the global trendline in Fig. 2b. The number of colonies per capita globally declined by 23.9% from 15.9 per 1000 population in 1961 to 12.1 per 1000 population in 2017. The decline occurred in all regions except in Asia and South America where small increases were experienced. Sharp declines were experienced in the late 1980’s to the early 1990’s, particularly in Europe and Oceania. The number of colonies per capita for these two regions started increasing in the late 2000s-early 2010s.

### Honey production

**Figure 3:**
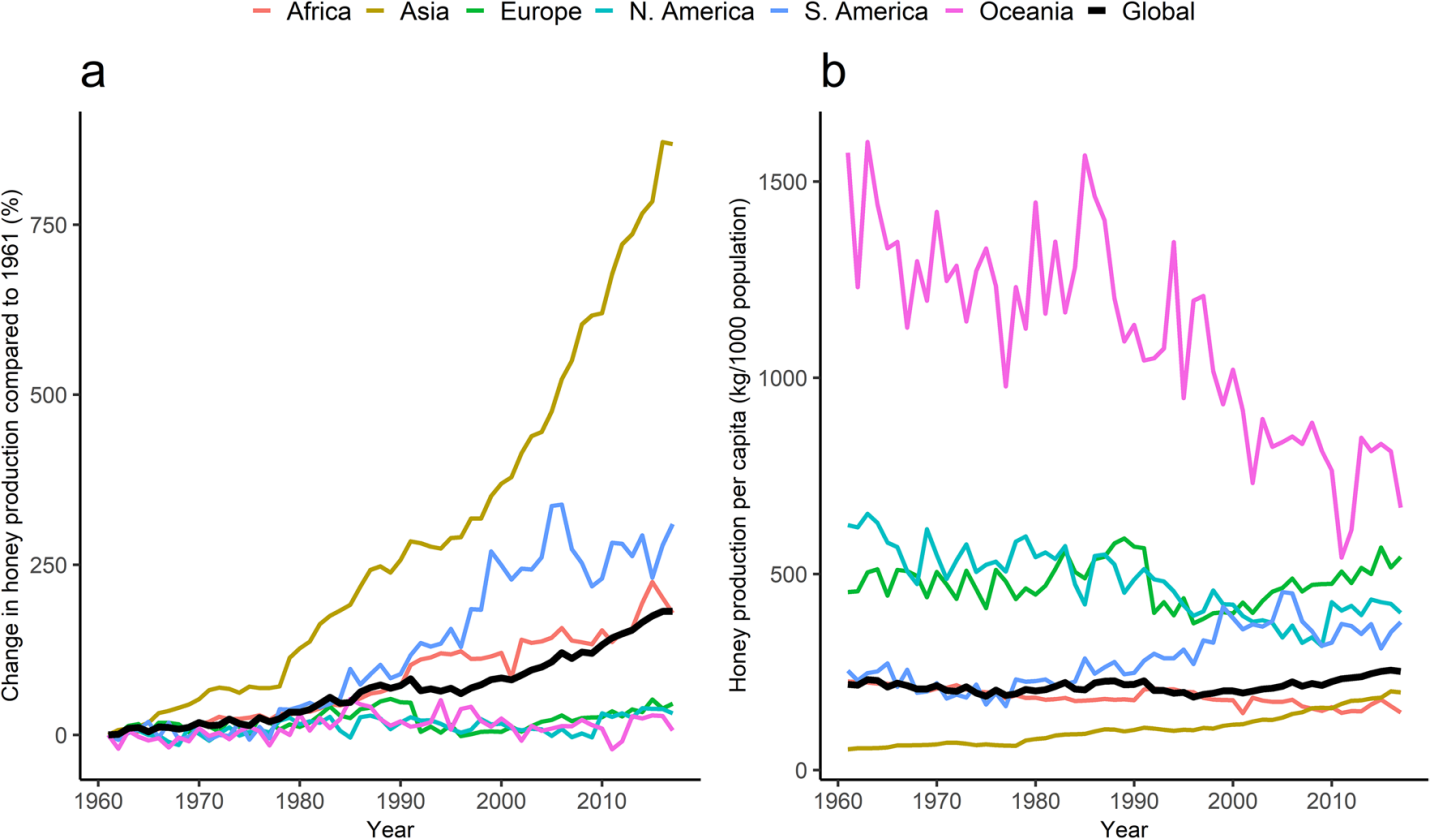
Regional annual honey production presented as percentage change compared to 1961 production (**a**) and production per capita (**b**), 1961–2017.

There was sustained increase in the global production of honey over the study period (Fig. 3a). This was influenced by the increases in Asia, South America and Africa. The amounts of honey produced in Europe, North America and Oceania remained similar to those of 1961 throughout the period of interest. Honey production per capita increased by 15.6% at the global level, from 218 kg per 1000 population in 1961 to 252 kg per 1000 population in 2017 (Fig. 3b). There was a remarkable decline in Oceania but less so in Africa, Europe and North America. Only Asia and South America had an upward trend in honey production per capita.

### Beeswax production

**Figure 4:**
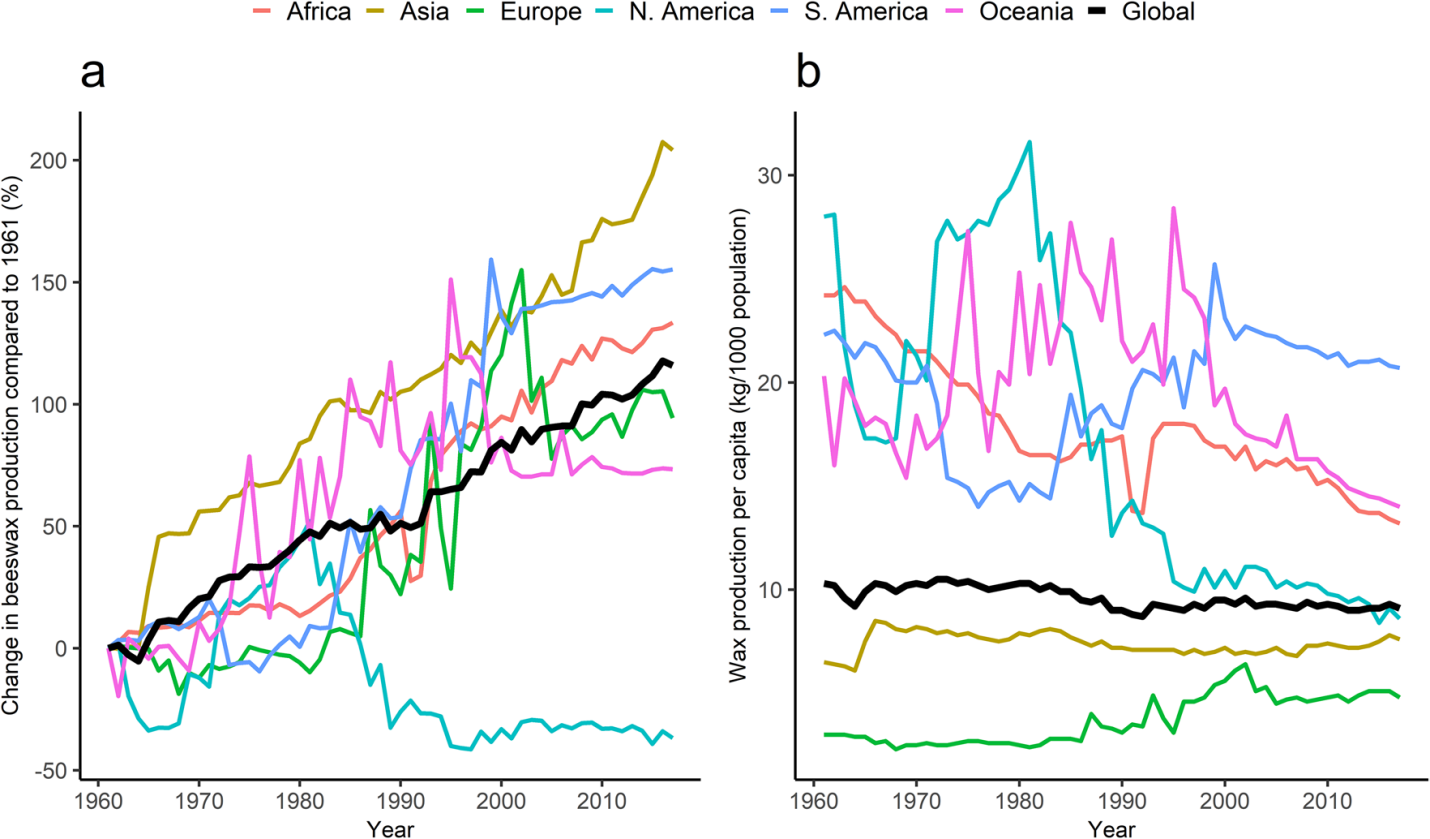
Regional annual beeswax production presented as percentage change compared to 1961 production (**a**) and production per capita (**b**), 1961–2017.

The global production of beeswax increased steadily during the study period under review (Fig. 4a). Production increased in all regions except North America where it declined sharply in early 1960s and again in the early 1980s to remain below the 1961 levels. Oceania beeswax production peaked in mid 1990s and levelled off after 2000 while in Europe production peaked in the early 2000s. Africa and Asia experienced the most steady and consistent production increases throughout the period. The global production of beeswax per capita decreased by 11.7% from 10.3 kg per 1000 population in 1961 to 9.1 kg per 1000 population in 2017 (Fig. 4b). Asia and Europe had the lowest beeswax production per capita throughout the period of interest. Africa had the most consistent decline in beeswax production per capita followed by North America after 1980. South America and Oceania also had downward trends after 2000.

### Honey yield

**Figure 5:**
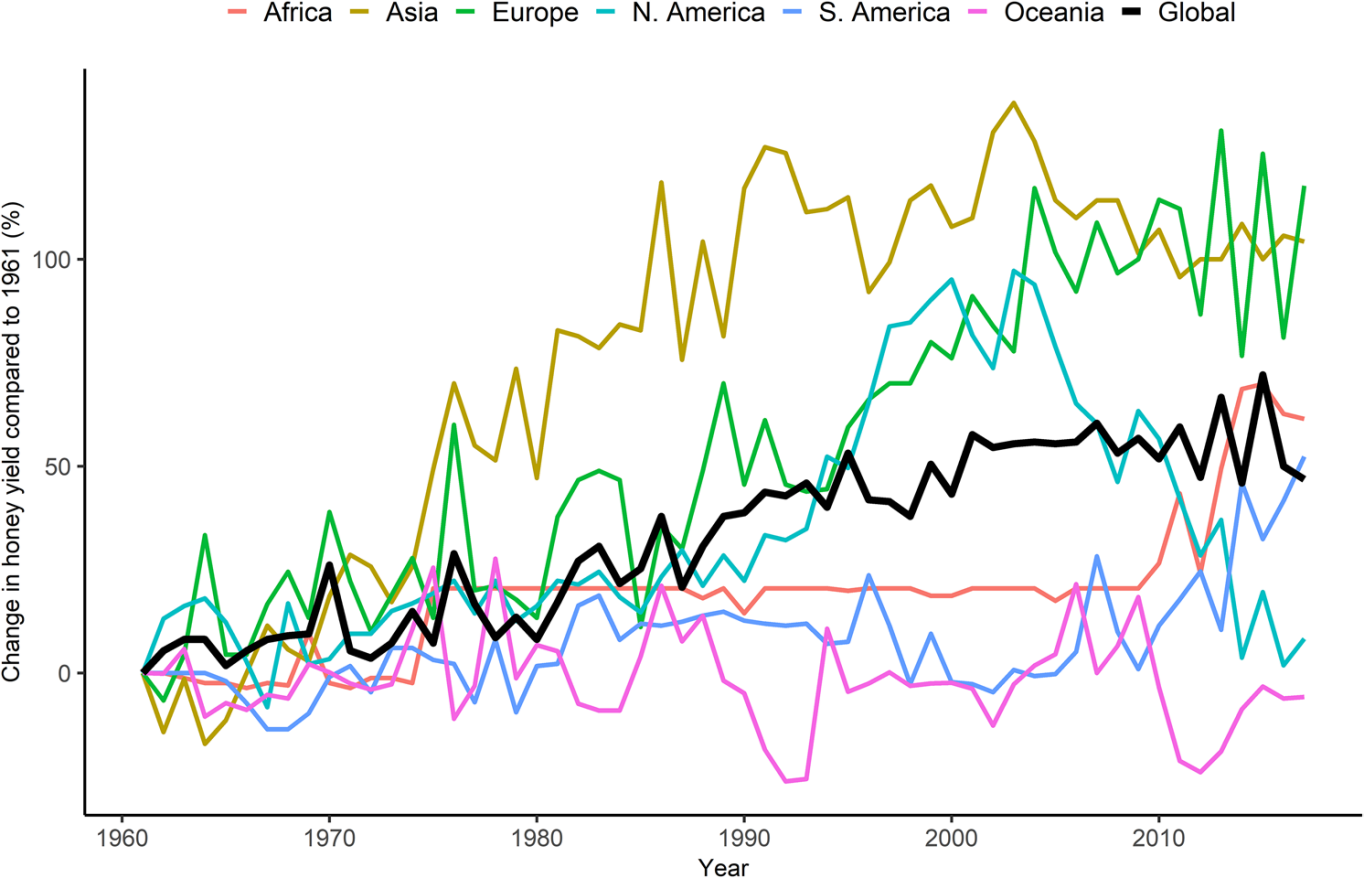
Percentage change in the median yield of honey per colony compared to 1961.

The yield of honey per colony improved globally by about 50% with the highest improvements in Asia and Europe where they peaked at over 100% between 1961 and 2017 (Fig. 5). In North America the yields peaked during early 2000s and then steadily dropped to reach the 1961 levels by the end of the study period. In Africa, the yields remained the same from the mid-1970s to early 2010’s, after which they increased. The yields in Oceania oscillated around those of 1961 similar to those of South America.

### Trends at country-level

**Figure 6:**
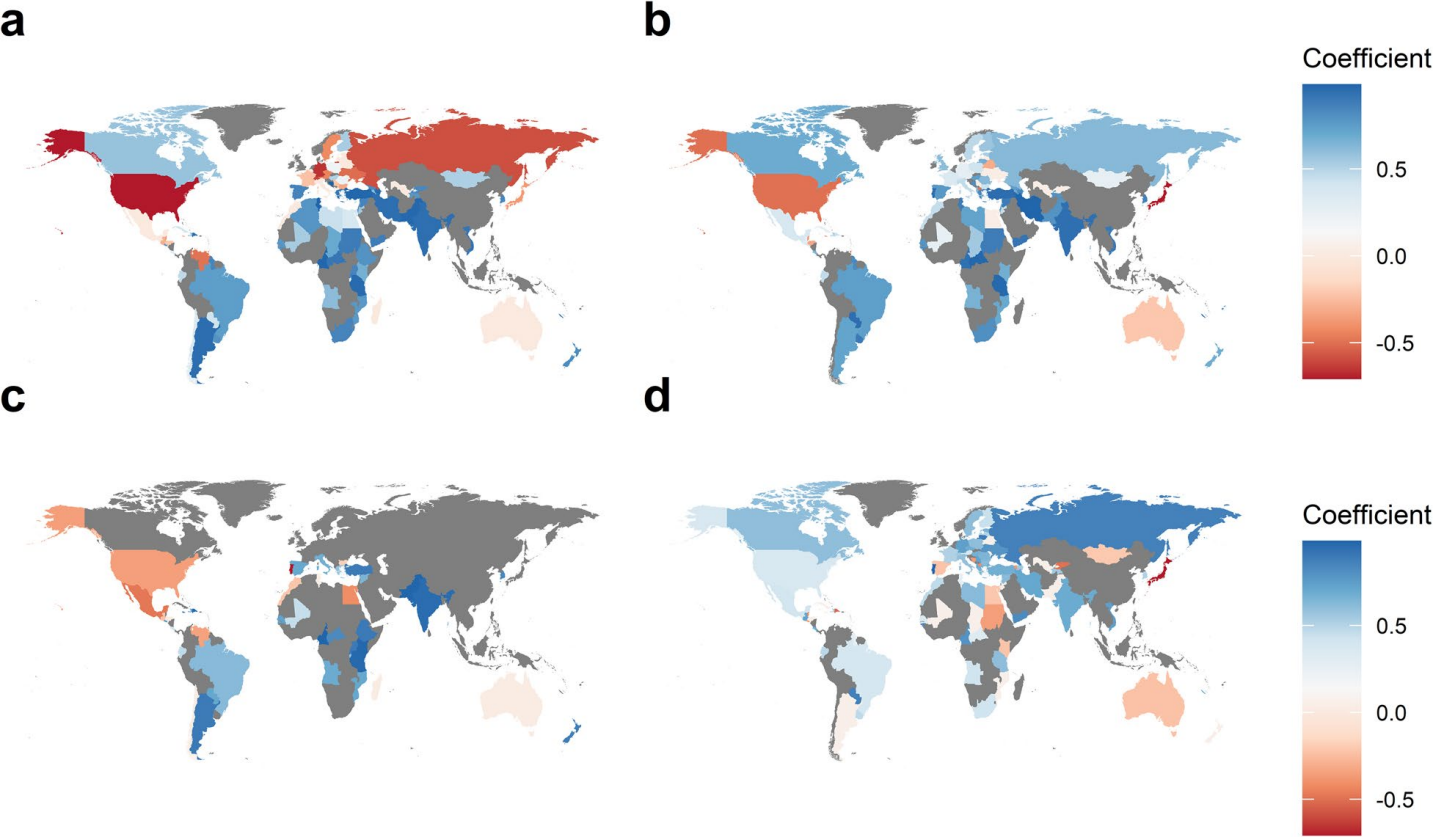
Heat maps showing monotonous increasing or decreasing trends in beekeeping variables. Monotonous changes in (**a**) the number of colonies, (**b**) honey productions, (**c**) wax productions, and (**d**) honey yield per colony between 1961 and 2017 are shown, where higher coefficients indicate more stable increases over time. Coefficients for countries shaded in dark were not computed due to missing data.

A country-level trend analysis suggested that regional trends shown above were also observable in most countries in each region (Fig. 6). Nevertheless, there were some exceptions. While the number of colonies and honey production monotonously decreased in the United States, those in Canada show an increasing trend. Similarly, there were increasing and decreasing trends for New Zealand and Australia, respectively. Many Asian countries showed increasing trends in the number of managed honey bee colonies, honey productions and honey yield per colony, but opposite trends were observable for Japan. There were notable mixed trends for honey yield per colony in Africa.

**Figure 7:**
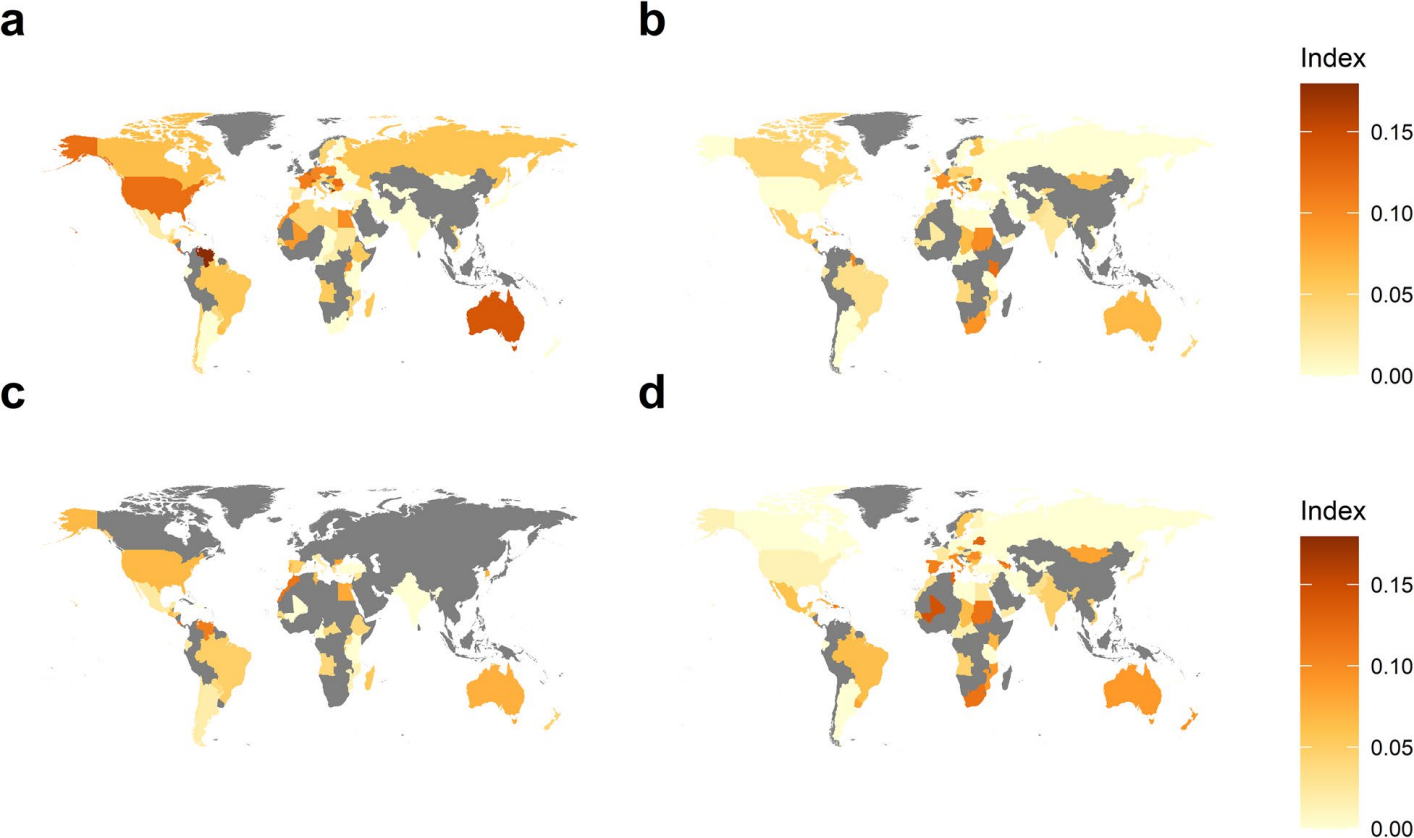
Heat maps showing the extent of anormal sudden drops in beekeeping variables. Indices representing the degree of anormal drops in (**a**) the number of colonies, (**b**) honey productions, (**c**) beeswax productions, and (**d**) honey yield per colony are shown. Higher indices represent the presence of more recent and frequent anormal drop events.

A further analysis was carried out to identify the extent of anormal sudden drops in the study variables (Fig. 7). It is evident that countries such as the United States, Venezuela, Australia and many European countries had more recent and frequent anormal drops in the number of colonies (Fig. 7a). Anormal sudden drops in honey productions observed in some southern European and African countries coincide with the drops in the honey yield per colony.

## Discussion

There has been speculation that the population of managed honey bees has declined worldwide. Our analysis shows that the global number of managed honey bee colonies and the production of honey and beeswax have increased in the last six decades. However, there were geographical variations in the trends at both the regional and country levels. A similar conclusion was drawn by a recent study which reported that honey bee populations were stable or increasing in three quarters of the countries surveyed^17^. These findings suggest that local factors at these levels were important determinants for changes occurring in beekeeping and production of bee products^18^. Such factors include habitat change^19^, pesticide usage^20^, pest infestation^21^, disease occurrence^22^, colony husbandry practices^23^ and climate change^22^. Commerce is another important factor as it drives decision-making at both the local and international levels. For instance, the demand for bee products or pollination services influences the direction of beekeeping in certain regions or countries such as predominantly honey-producing or pollination^24,25^. However, elucidation of these factors in relation to the observed beekeeping patterns is beyond the scope of the current study.

Regionally, the number of managed honey bee colonies increased in all but Europe and North America. Countries in the north of Europe appear to have experienced greater declines compared to those in the south. The downward trend in North America was influenced by the decline in the United States and Mexico while Canada had an upward trend. Many studies have documented the loss of colonies in these areas^23^ and have suggested that multiple factors are responsible for the loss of colonies ^26,27^. However, it is worth noting that heavy colony losses have occurred in history without bee populations completely disappearing^1,28^. Interpretation of colony losses should be different from losses of other livestock such as cattle that have an annual reproduction cycle. Instead, seasonal colony losses should be interpreted as turnovers as beekeepers can quickly replace them by either splitting the remaining colonies to create new ones or purchasing more bees for replenishment. Caron et al.^29^ reported that in 2008 and 2009 beekeepers in the Pacific Northwest replaced more colonies than they lost in the preceding winter. Perhaps this has helped Europe and North America experience relatively modest growth in managed colony populations in the last two decades, as shown by our analysis, even though they report high winter colony losses.

Whilst the absolute numbers of managed colonies increased over the study period globally, their estimates declined per capita. However, these could be inaccurate because there were larger amounts of data missing for the beekeeping variables compared to the human population data. It is also possible that the FAO dataset did not include data from hobbyists and small non-commercial beekeeping entities. Many national reports tend to have limited coverage of this category of the beekeeping industry. Additionally, our estimates were not based on the total bee population but that of managed honey bees, excluding non-managed bees. The size of the non-managed bee population and the associated trend is unknown. Therefore, caution should be exercised when interpreting estimates in the current study, particularly if extrapolated to a population that includes non-managed bees. Nevertheless, the estimates provide valuable insights in the long-term trends for the managed honey bee population.

The declining trend in the number of managed colonies per capita suggests that the global honey bee population grew slower than that of humans during the period under study. This means that as we go into the future, a given population of honey bees will be serving a larger human population in terms of supplying bee products and providing pollination services. As affluence of the human population also grows, the impact on pollination-dependent agriculture could be profound as managed honey bees are the major pollinators, but by no means the only ones. Previous research has highlighted similar concerns^30^. The shortage of pollinators could result in increased cost of pollination services, suboptimal production of pollination-dependent crops and higher prices of the affected crops. A combination of these factors could affect food security leading to nutritional deficits in some regions of the world.

The trends for beeswax production were similar to those for the managed colony population. The total global production of beeswax increased while production per capita decreased. The global reduction of beeswax production per capita could be related to a similar decline in the number of colonies per capita during the period of interest. However, other factors could also have played a role such as reduced market demand due to changes in the industrial usage of beeswax. For instance, the replacement of beeswax with cheaper petroleum-based products in some industrial processes. The factors could be different in North America as the production trends were remarkably different from those of other regions. A corresponding decline of managed honey bee colonies per capita may have been a contributing factor in this region. It is worth noting that beeswax production had the largest amount of missing data which could have affected the accuracy of our estimates. However, they provide useful broad scale patterns.

The trends for global honey production were different from those of managed colonies and beeswax production in that both total production and production per capita increased over the study period. The increase of global honey production per capita despite a corresponding decrease in the number of colonies per capita suggests an improvement in the efficiency of producing honey. This is supported by improved yields of honey per colony globally over the same period. However, other factors could also have contributed to the trends such as improved beekeeping husbandry practices and increased or better floral sources for the bees. The introduction of *A. mellifera* in Asia to replace the indigenous *A. cerana* for honey production could partly explain the improvement of both the volume produced and yields per colony in that region. Kosaka et al.^31^ reported that *A. mellifera* now supersedes *A. cerana* which was once dominant in Japan and South Korea. This has resulted in improved honey production in the two countries. It is also possible that the volume of honey produced globally is artificially inflated through adulteration. The characterisation of honey as a high value natural foodstuff with medicinal or health benefits to humans has made it a target of adulteration^32,33^.

Regionally, Asia and Europe experienced the highest improvements in yields per colony. Oceania oscillated between improving and getting worse compared to the 1961 yields. In Africa and South America, the yields remained relatively stable for many years but improved towards the end of the study period. The data available for the current study are inadequate to provide conclusive explanation for these variations. However, some key variables that influence the performance of beekeeping have changed over the study period in some regions of the world. For instance, a recent report indicated that honey bees now have more temperature-eligible flight hours compared to 40 years ago due to warming of the climate^34^. This suggests that areas previously unsuitable for beekeeping because of low temperatures can now support honey production. It also means that honey bees may now have more time to forage resulting in higher honey yields and production. As noted earlier, the introduction of *A. mellifera* in Asia has led to improvements to production and yields in that region.

In conclusion, evidence suggests that the number of managed honey bee colonies as well as the production of honey and beeswax globally have increased since the early 1960s. However, the number of colonies and beeswax production per capita declined. This means a larger human population is now reliant on the same the number of honey bee colonies for their services than before. It is likely the dependence will only exacerbate as the level of affluence increases worldwide. Short-term effects such as political change, adverse seasonal weather events, acute environmental mismanagement and inadequate disease control could explain sudden drops in the number of colonies^4^ or production of honey and beeswax in some regions. For instance, the decline in the number of managed colonies in Europe during the 1990s was attributed to political and economic impacts of the dissolution of the Soviet Union^35^. Sudden drops in numbers of colonies or production do not appear to be long-lasting. The varied challenges in different regions suggest that locally driven solutions are important when developing strategies and measures for intervention. Resilience in beekeeping could be enhanced by improving husbandry practices, genetics of honey bees, agroecology management, landscape restoration and control of pests and diseases. Promoting the usage of bee products as natural and sustainable products could also help stimulate growth in the beekeeping industry.

## Materials and methods

FAO collects data from member countries using a questionnaire sent annually to gather information for the three preceding years^36^. The quality of the data is likely to be variable depending on the capability and standards of each country to collect and manage data. Details such as the species of honey bees, type of honey or number of beekeepers are not recorded in the FAO database. However, it is safe to assume that *A. mellifera* is dominant as it produces more honey than other bee species and is favoured by beekeepers. The use of other species such as *A. cerana* is limited to their native regions, particularly Asia^2,37^.

We used three variables to assess how the global beekeeping industry changed over time: number of colonies, honey production and beeswax production. As a derivative, honey yield was used to assess change in production efficiency. We standardised the beekeeping variables using human population, allowing us to assess the expansion of the beekeeping industry against human population growth. R version 3.6.3^38^ was used conduct all the analyses and produce the Figures in the current report.

### Datasets

We retrieved annualised country-level beekeeping data for the period 1961–2017 from FAO’s public facing website^39^. For region-level analysis, these data were aggregated into six regions: Africa, Asia, Europe, North America, South America and Oceania. Similarly, we obtained annualised country-level human population data from a website maintained by Global Change Data Lab and University of Oxford^40^ and aggregated them into the six regions.

### Region-level analysis

For study variable *x* (colonies, honey production, beeswax production, honey yield) in region *j* (Africa, Asia, Europe, North America, South America, Oceania, Global), the percentage change for year *t* (1961–2017) compared to 1961 was calculated using the formula:

1 $$\Delta x_{jt} = 100 \times \left( {x_{jt} - x_{j1961} } \right)/x_{j1961}$$

Variable per capita *y* (per 1000 population) in region *j* and year *t* was calculated using the formula:

2 $$y_{jt} = 1000 \times \left( {x_{jt} /p_{jt} } \right)$$

where *p* is the human population.

### Country-level analysis

To illustrate the trends at a finer resolution, we carried out two country-level analyses. First, we evaluated whether each study variable had a monotonous, either increasing or decreasing, trend over time. For each country, Kendall correlation coefficient was computed for data points between 1961 and 2017. A coefficient close to 1 indicates that a given study variable had been continuously increasing over the study period, whereas − 1 indicates a continuous decrease. Second, we aimed to identify countries, if any, where anormal drops have been observed for the study variables. We applied the robust peak-detection algorithm based on z-score^41,42^ to the time-series data of each of the four study variables. In short, we calculated a moving average of a given variable over five consecutive data points (i.e. five years) and considered a new data point to be anormal if this data point was three standard deviations away from the moving average. Anormal data points were assumed to have 50% of weight as normal data points in calculating the moving average and standard deviation so that the detection algorithm remains stable regardless of the number of abnormalities. We calculated an anormal drop index *I*_*j*_ for country *j* as follows:

3 $$Ij = \sum\nolimits_{t = 1966}^{{t_{end} }} {Ind_{t} \times w_{t} }$$

where *t* represents a year, *t*_*end*_ is the last data point (i.e. 2017), *Ind*_*t*_ is an indicator variable which takes 1 if an anormal drop was observed in year *t* (otherwise takes 0), and *w*_*t*_ is a weight variable which was calculated using the formula:

4 $$w_{t} = \frac{{\log \left( {t_{end} + 2 - t} \right)}}{{\mathop \sum \nolimits_{t = 1966}^{{t_{end} }} \log \left( {t_{end} + 2 - t} \right)}}$$

Therefore, more recent anormal drop events have larger contributions to *I*_*j*_, which takes 1 when anormal drop events occurred every year between 1966 and 2017. Note that *t* starts from 1966 because this algorithm requires at least five previous data points. Calculated indices were visualised using a heat map.

For each study variable, we carried out country-level analyses for countries that have no missing data except for countries that were part of the Soviet Union and had no missing data since 1992. Some countries in each region had missing data for the period of interest. Among the study variables, the number of colonies had the most abundant data across regions; approximately 80% of the countries in each region had complete datasets. The completeness of honey production data was more heterogeneous across regions than other variables. Oceania had 9 out of 10 countries with complete honey production dataset, 61.5% of African countries and 60% of South American countries had complete data for this variable. Beeswax production data were the least complete, with full datasets being available for 14.7% of European, 20% of Oceanian, and 24% of Asian countries. For human population data, South America had one (out of 10) and Africa had two (out of 26) countries with missing data. More than 15% of countries in other regions had missing data, with Oceania having the highest proportion of missing (20%).

## Supplementary Information

Supplementary Information.

## Data Availability

The data on the number of colonies, honey production, honey yield and beeswax production are available on the FAO website at https://www.fao.org/faostat/en/#data/QCL The human population data can be found on this website maintained by Global Change Data Lab and University of Oxford at https://ourworldindata.org/grapher/global-and-regional-population-estimates-us-census-bureau-vs-un.
