# Supplementary material for: Uptrend in global managed honey bee colonies and production based on a six-decade viewpoint, 1961–2017
Source: Sci Rep. 2022 Dec 9;12:21298. doi: 10.1038/s41598-022-25290-3 (PMC9734161; doi:10.1038/s41598-022-25290-3)
Supplement: Supplementary file 1 — Supplementary Information. [file 41598_2022_25290_MOESM1_ESM.docx]

**Table S1.** Country-level average numbers of colonies and volumes of honey and beeswax produced per annum over the five-year 2013-2017, arranged in descending order of the value. Estimates are accompanied with the 95% confidence interval (CI) and expressed as percentage of the global total.

| **1961-196.** |  |  |  | **2013-2017** |  |  |
| --- | --- | --- | --- | --- | --- | --- |
| **Country** | **Quantity**  **(95% CI)** | **%** |  | **Country** | **Quantity**  **(95% CI)** | **%** |
|  |  |  |  |  |  |  |
| Colonies (000,000) | | | | | | |
| Global | 49.8 (94.5; 101.0) | 100.0 |  | Global | 88.5 (85.5; 91.5) | 100.0 |
| Soviet Union | 10.3 (9.9; 10.8) | 20.8 |  | India | 11.9 (11.6: 12.1) | 13.4 |
| United States | 5.4 (4.9; 5.8) | 10.8 |  | China | 9.1 (9.0; 9.2) | 10.3 |
| India | 5.3 (4.5; 6.0) | 10.6 |  | Turkiye | 7.5 (6.7; 8.2) | 8.4 |
| China | 3.5 (3.4; 3.7) | 7.1 |  | Iran | 7.0 (6.7; 7.4) | 8.0 |
| Ethiopia | 2.5 (2.4; 2.6) | 5.0 |  | Ethiopia | 5.8 (5.3; 6.4) | 6.6 |
| Germany | 1.8 (1.6; 1.9) | 3.6 |  | Russia | 3.4 (3.3; 3.5) | 3.8 |
| Turkiye | 1.6 (1.5; 1.7) | 3.1 |  | Argentina | 3.0 (3.0; 3.0) | 3.4 |
| Mexico | 1.5 (0.8; 2.1) | 2.9 |  | Tanzania | 2.9 (2.9; 3.0) | 3.3 |
| Poland | 1.2 (1.2; 1.2) | 2.4 |  | United States | 2.7 (2.6; 2.8) | 3.0 |
| Angola | 1.0 (1.0; 1.0) | 2.0 |  | Spain | 2.7 (2.5; 2.9) | 3.0 |
|  |  |  |  |  |  |  |
| Honey production (0,000 tonnes) | | | | | | |
| Global | 71.0 (66.9; 75.2) | 100.0 |  | Global | 183.0 (173.0; 193.0) | 100.0 |
| Soviet Union | 20.7 (19.4; 22.0) | 29.1 |  | China | 50.7 (44.9; 56.4) | 27.6 |
| United States | 12.5 (11.3; 13.6) | 17.5 |  | Turkiye | 10.5 (9.6; 11.4) | 5.7 |
| China | 5.7 (5.4; 6.1) | 8.1 |  | Canada | 8.9 (7.9; 9.9) | 4.9 |
| Mexico | 2.9 (2.4; 3.4) | 4.1 |  | Iran | 7.3 (6.8; 7.7) | 4.0 |
| Argentina | 2.2 (1.7; 2.7) | 3.0 |  | United States | 7.2 (6.5; 7.9) | 3.9 |
| India | 2.1 (1.8; 2.4) | 3.0 |  | Russia | 6.9 (6.5; 7.4) | 3.8 |
| Angola | 2.0 (2.0; 2).0 | 2.8 |  | Argentina | 6.8 (5.6; 8.0) | 3.7 |
| Australia | 1.9 (1.6; 2.2) | 2.6 |  | Ukraine | 6.6 (5.9; 7.2) | 3.6 |
| Canada | 1.8 (1.4; 2.2) | 2.5 |  | India | 6.3 (6.1; 6.6) | 3.5 |
| Ethiopia | 1.5 (1.4; 1.6) | 2.1 |  | Mexico | 5.7 (5.2; 6.3) | 3.1 |
|  |  |  |  |  |  |  |
| Beeswax (000 tonnes) | | | | | | |
| Global | 31.6 (30.2; 32.9) | 100.0 |  | Global | 67.2 (65.0; 69.4) | 100.0 |
| India | 10.5 (9.1; 12) | 33.3 |  | India | 24.2 (23.0; 25.4) | 76.8 |
| Mexico | 3.6 (2.1; 5.2) | 11.5 |  | Ethiopia | 5.4 (5.1; 5.7) | 17.0 |
| United States | 2.2 (2.1; 2.2) | 6.8 |  | Argentina | 4.9 (4.8; 4.9) | 15.4 |
| Angola | 2.0 (2.0; 2.0) | 6.3 |  | Turkiye | 4.5 (4.1; 5.0) | 14.3 |
| Ethiopia | 1.5 (1.4; 1.6) | 4.7 |  | South Korea | 3.7 (3.1; 4.3) | 11.8 |
| Argentina | 1.3 (1.3; 1.4) | 4.2 |  | Kenya | 2.5 (2.5; 2.5) | 8.0 |
| Brazil | 1.3 (1.2; 1.4) | 4.0 |  | Angola | 2.3 (2.3; 2.3) | 7.3 |
| Turkiye | 1.0 (0.9; 1.1) | 3.1 |  | Tanzania | 1.9 (1.9; 1.9) | 5.9 |
| Madagascar | 0.9 (0.9; 0.9) | 3.0 |  | Mexico | 1.8 (1.5; 2.0) | 5.6 |
| Portugal | 0.6 (0.6; 0.6) | 1.9 |  | Brazil | 1.7 (1.7; 1.8) | 5.4 |
